# Food-chain spirochetes: a unified hypothesis for Parkinson’s disease and dementia risk

**DOI:** 10.3389/fnagi.2026.1798699

**Published:** 2026-05-07

**Authors:** Linda M. Sweeney

**Affiliations:** Independent Researcher, Woodland, WA, United States

**Keywords:** biofilm, dementia, gut-brain axis, microbial persistence, Parkinson’s disease, poultry processing, spirochete

## Abstract

Parkinson’s disease (PD) and dementia have risen markedly in many regions. While aging, genetics, toxicants, and protein misfolding explain key aspects of pathology, they do not fully account for synchronized temporal trends or pronounced regional heterogeneity in recent increases. This hypothesis and theory article proposes that persistent microorganisms in industrial food systems—particularly biofilm-forming spirochetes in high-throughput poultry processing—may represent an under-recognized upstream contributor via the gut–brain axis. Analysis of Global Burden of Disease and FAOSTAT data reveals a population-level temporal alignment between rising PD incidence and poultry consumption in the United States and China, with a multi-year lag consistent with prodromal disease phases. In contrast, countries with differing sanitation and feed-chain policies (e.g., Israel’s kosher salting and Germany’s oxidative/thermal methods) show more stable neurodegenerative trends despite high poultry intake. These patterns suggest that sanitation chemistry, processing throughput, and animal-protein recycling may influence long-term microbial exposure risk. The proposed model integrates infection, gut–brain signaling, and protein-misfolding pathways by positioning chronic microbial persistence as a plausible initiating or amplifying factor. Supporting observations include spirochete detection in neurodegenerative tissue, biofilm adaptation under disinfection stress, and experimental evidence linking intestinal bacteria to *α*-synuclein pathology. Testable predictions include detection of resilient spirochetes or related taxa in post-sanitation products and higher microbial signatures in relevant patient samples.

## Introduction

Parkinson’s disease (PD) and dementia together represent a growing global public health burden, with rising incidence, prevalence, and societal cost documented across regions in recent decades (Steinmetz et al., 2024; Food and Agriculture Organization of the United Nations, 2024; World Organization for Animal Health (WOAH, formerly OIE), 2011; Israeli Ministry of Health, n.d.; Yakobson et al., 2014). In the United States alone, dementia costs approximately $360 billion annually, while Parkinson’s disease adds an estimated $52 billion, together exceeding $420 billion (Alzheimer’s Association, 2024; Parkinson’s Foundation, 2024). Despite decades of research, the factors underlying recent increases in these disorders remain incompletely explained. The parallel rise in PD and dementia incidence since the late 1990s (Steinmetz et al., 2024) suggests that modern environmental factors may contribute.

Parkinson’s disease and dementia are complex, multifactorial conditions shaped by aging, genetic susceptibility, environmental exposures, and biological processes including inflammation and protein misfolding. The present hypothesis does not seek to replace these established models but instead explores whether persistent microbial exposure within modern food systems may represent an additional upstream contributor acting in concert with these factors. In this paper, ‘dementia’ is used as a broad clinical category that includes Alzheimer’s disease and related neurodegenerative conditions unless otherwise specified.

### Limitations of current theories

Genetic susceptibility, aging, pesticide exposure (Van Maele-Fabry et al., 2012), and spontaneous protein misfolding (Prusiner, 1998)—each explain aspects of the pathology but do not account for the synchronized global timing or regional differences in disease trajectories. The classical prion hypothesis attributes certain neurodegenerative disorders to self-propagating misfolded proteins (Prusiner, 1998); however, similar aggregates are also observed in chronic inflammatory and microbial conditions (Allen et al., 2016; Miklossy, 2011; Miklossy, 2015), suggesting they may, in some contexts, reflect downstream processes rather than solely acting as initiating factors. These approaches do not fully explain the epidemiologic patterns examined in subsequent sections.

### The prion paradigm revisited

The prion theory describes a protein-templating mechanism in which misfolded proteins propagate by conformational change rather than conventional replication (Prusiner, 1998). This framework does not depend on cross-species transmissibility or an identified initiating exposure, and spontaneous forms of prion disease are well recognized (Prusiner, 1998). The present hypothesis does not challenge prion-like mechanisms in neurodegeneration; rather, it asks whether chronic microbial or inflammatory exposures could act as one upstream factor within broader pathways involving protein misfolding, neuroinflammation, and host susceptibility (Allen et al., 2016; Miklossy, 2011; Miklossy, 2015; Brundin et al., 2010). In parallel, microbial contributors such as spirochetes were not routinely investigated in neurodegenerative tissue using methods sensitive to low-abundance organisms during the period when prion models became established.

Modern PCR and immunohistochemical studies have identified *Treponema* DNA in Alzheimer’s brain tissue (Riviere et al., 2002), and spirochetes have been visualized within *β*-amyloid plaques in neuropathologic studies (Miklossy, 2011; Miklossy, 2015). *Brachyspira* has been detected in poultry gut (Burch et al., 2006), underscoring its relevance as a food-chain exposure, particularly given the capacity of biofilm-associated organisms to persist under certain disinfection conditions and adapt to sublethal environmental stressors (Bridier et al., 2011; Giaouris et al., 2014; Calabrese and Mattson, 2017). Intestinal and other host-associated bacteria have been proposed to influence *α*-synuclein pathology through gut–brain signaling and inflammatory mechanisms (Sampson et al., 2016). Unlike prions, spirochetes are living organisms capable of persistence, host colonization, and sustained inflammatory signaling, making them biologically plausible as an upstream contributor to misfolding-associated cascades (Allen et al., 2016; Miklossy, 2011; Miklossy, 2015; Brundin et al., 2010).

### A new ecological hypothesis

This paper explores whether persistent microorganisms in industrial food systems may represent an ecological link between environmental change and neurodegeneration. Biofilm-forming spirochetes and other anaerobes in processing environments could act as infectious stimuli contributing to neurodegenerative processes. These organisms can persist under certain disinfection conditions and, through food handling or ingestion, interact with the gastrointestinal environment, with the potential to influence inflammatory pathways via the gut–brain axis (Sampson et al., 2016; Foster and McVey Neufeld, 2013; Cryan and Dinan, 2012).

Spirochetes have been observed in patterns partially consistent with selected aspects of Koch’s postulates and Hill’s criteria, although definitive proof remains to be established (Miklossy, 2011). Miklossy’s (2011, 2015) studies, demonstrated *Treponema* within *β*-amyloid plaques and cortical tissue in neurodegenerative brains—histopathologic evidence supporting further investigation. Experimental models further show that intestinal bacteria can initiate *α*-synuclein pathology and motor impairment, supporting the biological plausibility of gut–brain mechanisms (Sampson et al., 2016).

### Industrial context and global timing

The late-20th-century industrialization of poultry processing created ecological conditions favoring persistent microbial survival—accelerated line speeds, organic-rich environments, and reliance on high-volume chemical disinfection systems (National Chicken Council, n.d.; USDA Food Safety and Inspection Service, 1996; USDA Food Safety and Inspection Service, 2023). Beginning in the late 1990s and early 2000s, poultry-processing systems—particularly in the United States—expanded production and adopted progressively faster line speeds in response to rising demand (National Chicken Council, n.d.; USDA Food Safety and Inspection Service, 1996). Although throughput limits differ by country, the broader trend toward higher line speeds and mechanization is well documented in FAO production statistics (Food and Agriculture Organization of the United Nations, 2024) and U. S. federal inspection reports (USDA Food Safety and Inspection Service, 2023).

This global acceleration provides important context for the broadly parallel rise in neurodegenerative-disease burden observed in subsequent decades, consistent with long-term trends reported in GBD 2016 analyses (GBD 2016 Parkinson’s Disease Collaborators, 2018).

Regional contrasts further reinforce this pattern. Some countries have shown more stable neurodegenerative-disease trajectories despite substantial poultry consumption, suggesting that differences in food-system practices and environmental factors may contribute to variation in long-term exposure risk.

### Objectives of this paper

1. Present global and regional evidence describing how PD and dementia incidence corresponds with changes in poultry production and processing systems.
2. Integrate mechanistic pathways through which persistent microbial exposure could contribute to neuroinflammatory and protein-misfolding cascades.
3. Compare sanitation strategies across regions (e.g., chemical versus non-chemical approaches such as oxidative, UV, or salt-based methods) to explore modifiable environmental factors.
4. Outline surveillance and clinical or epidemiologic approaches to support hypothesis testing.

Together, these aims support a testable model integrating multiple theories within an ecological context, in which infection may precede or interact with protein misfolding and disease processes.

### Global patterns

Global surveillance data show that Parkinson’s disease incidence and poultry consumption have followed broadly similar temporal patterns since the early 1990s (Steinmetz et al., 2024; Food and Agriculture Organization of the United Nations, 2024). This period overlaps with a worldwide acceleration in poultry-processing throughput—including higher line speeds, expanded slaughter capacity, and increased chemical-based sanitation—adopted across multiple countries between the mid-1990s and early 2000s (USDA Food Safety and Inspection Service, 1996; USDA Food Safety and Inspection Service, 2023; GBD 2016 Parkinson’s Disease Collaborators, 2018; Balash et al., 2025).

Where national incidence data are unavailable or unstable, prevalence or modeled burden estimates are used for longitudinal comparison, with trends interpreted directionally rather than as absolute risk measures. Initial analyses examined other animal-protein categories, including beef and pork, given longstanding hypotheses linking red meat or saturated fat to neurodegenerative risk. However, no comparable temporal pattern was identified for beef or pork consumption. Poultry emerged as the most internally consistent ecological signal in this analysis.

**Figure 1:**
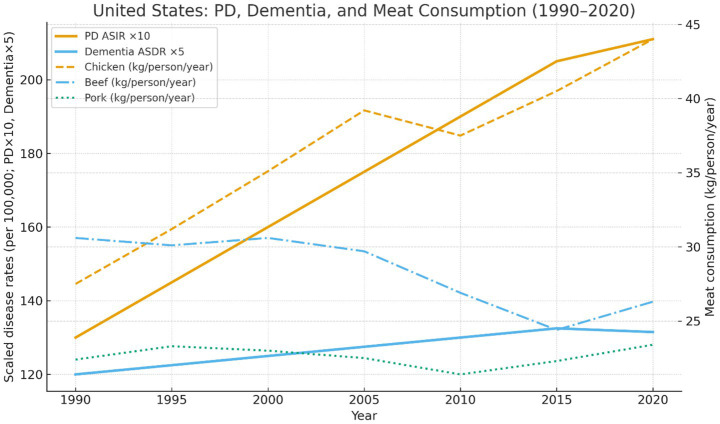
United States trends in Parkinson’s-disease age-standardized incidence rate (ASIR), dementia age-standardized death rate (ASDR), and per-capita chicken, beef, and pork consumption, 1990–2020 (5-year intervals). Disease rates are drawn from GBD 2021 (Steinmetz et al., 2024) and meat-supply data (kg/person/year) from FAOSTAT 2024 (Food and Agriculture Organization of the United Nations, 2024). PD ASIR increased by approximately 60% and showed a broadly similar upward trend to chicken consumption, whereas beef and pork intake remained relatively stable or declined over the same period. Disease rates (PD × 10, dementia ×5) are plotted on the left axis and meat consumption on the right axis, with scaling applied for visual comparison of temporal trends and not intended to imply equivalence of magnitude across variables. Dementia age-standardized death rate (ASDR) is used as a longitudinal indicator of disease burden due to limitations in consistent incidence data across the full period.

Figure 1 shows U. S. trends in Parkinson’s-disease incidence and per-capita meat consumption, 1990–2020.

**Figure 2:**
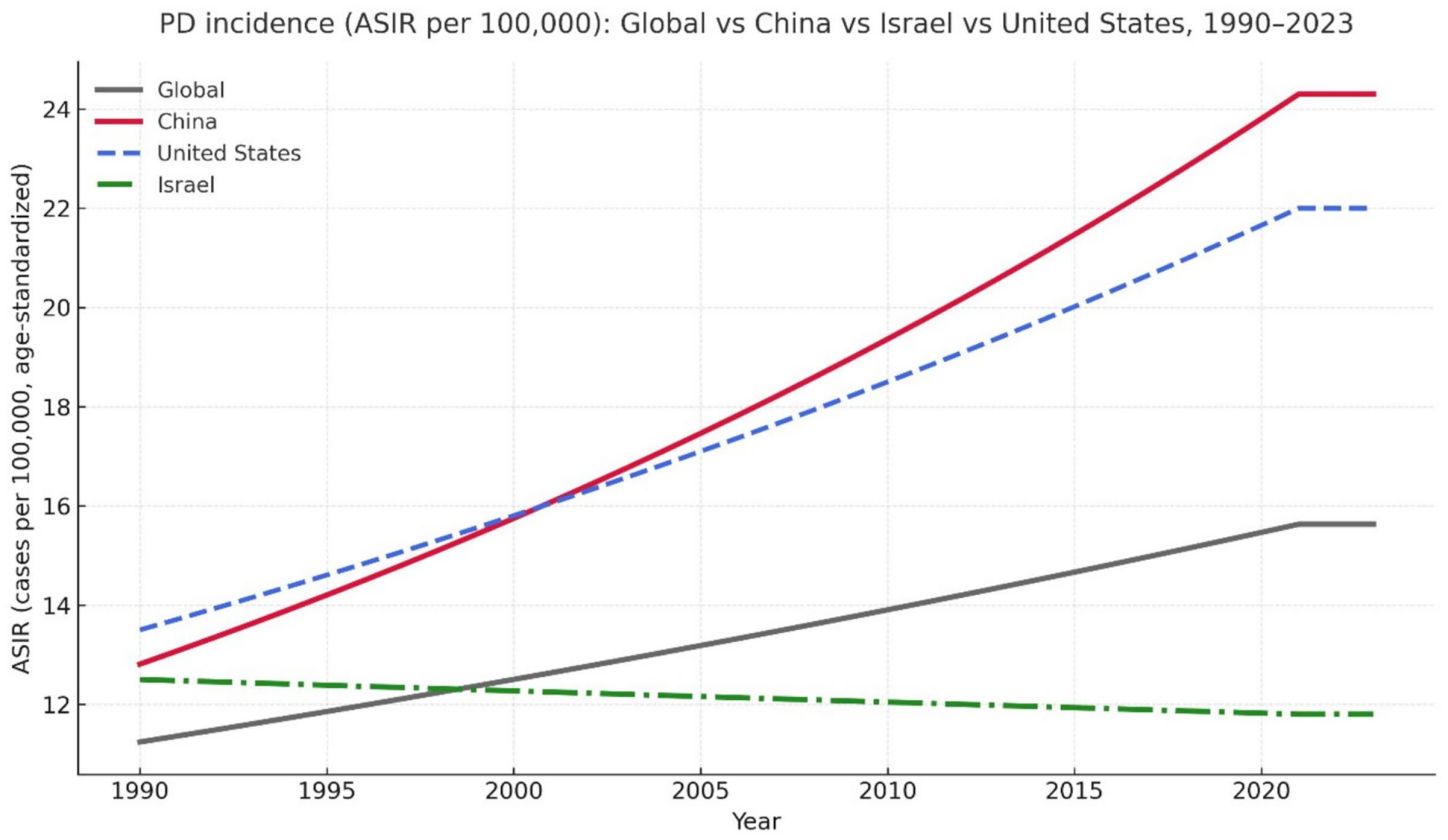
Age-standardized Parkinson’s-disease incidence rates (ASIR) for the United States, China, Israel, and global estimates. Data source: IHME Global Burden of Disease 2021 (Steinmetz et al., 2024). Values represent age-standardized incidence per 100,000 population.

Geographic comparisons show that long-term Parkinson’s-disease trends differ across countries with distinct processing environments and regulatory approaches (see Figure 2), highlighting geographic variation.

Recent analyses, including German administrative data, show a modest decline in age-standardized prevalence of Parkinson’s disease (0.38% → 0.29%, 2017–2022) and dementia (≈3.4% → 2.8% among adults ≥40 years) (Fink et al., 2025; Rommel et al., 2025a, b). Although based on prevalence rather than incidence, these claims-based estimates align with Germany’s broader stability in neurodegenerative-disease indicators and with global GBD 2021 trends (Steinmetz et al., 2024).

Detailed comparisons of sanitation practices, feed policies, and throughput conditions are examined in the following section. Countries were selected based on the availability and consistency of long-term epidemiologic and food-system data, together with differences in processing conditions, sanitation practices, and feed-chain policies.

The United States and China represent high-throughput processing systems in which intra-species recycling of animal by-products remains permitted, maintaining closed-loop pathways within the food chain. In contrast, Israel and Germany provide informative comparisons due to differences in feed-chain controls, sanitation practices, and antimicrobial use. National data from Israel indicate stable to modestly changing dementia incidence in EHR-based analyses (Lutski et al., 2022), whereas China has experienced substantial increases in dementia burden over recent decades (Hou and Yang, 2025).

Germany’s greater reliance on oxidative and thermal sanitation methods, along with lower throughput conditions and higher-temperature rendering, may reflect reduced microbial viability and persistence (Bridier et al., 2011; Giaouris et al., 2014; Calabrese and Mattson, 2017). Israel’s feed restrictions reflect broader principles established following the bovine spongiform encephalopathy crisis, including prohibitions on mammalian protein in animal feed and strengthened feed-chain controls (World Organization for Animal Health (WOAH, formerly OIE), 2011; Israeli Ministry of Health, n.d.; Yakobson et al., 2014).

### Regional contrasts

Table 1 summarizes key differences in poultry-system hygiene and neurodegenerative trends across selected countries and regions.

**Table 1:** Poultry-system hygiene and Parkinson’s-disease incidence trends (1990–2021).

Country/Region	Hygiene strategy	PD Trend (EAPC/ASIR)	Dementia Trend (Prevalence/EAPC)	Data source
Germany	Common use of peracetic-acid/hot-water sanitation; restrictive feed controls; comparatively lower antibiotic use	Modest decline in claims-based PD prevalence (0.38% → 0.29%, 2017–2022) (Rommel et al., 2025a)	Decline in claims-based dementia prevalence (~3.4% → 2.8% among adults ≥40, 2017–2022) (Rommel et al., 2025b)	Rommel et al. (2025a,b)
Israel	Kosher salting and rinsing practices; restrictions on animal-protein feed post BSE-related reforms	Stable to slightly declining PD incidence in national surveillance reports (Balash et al., 2025)	Stable to modestly changing dementia incidence in EHR-based national studies (Lutski et al., 2022)	World Organization for Animal Health (WOAH, formerly OIE), (2011), Israeli Ministry of Health (n.d.), Yakobson et al. (2014) Balash et al. (2025) Lutski et al. (2022)
United States / China	Common use of chemical antimicrobial disinfection; large-scale, high-throughput processing	Long-term increase in PD incidence (positive ASIR trend, 1990–2021) (Steinmetz et al., 2024)	Increasing modeled dementia burden (substantial modeled rise, 1990–2019) (Steinmetz et al., 2024)	Steinmetz et al. (2024)

Data sources: IHME Global Burden of Disease 2021 (Steinmetz et al., 2024); RKI Journal of Health Monitoring (2025) (Rommel et al., 2025a; Rommel et al., 2025b); industry and national agricultural data (National Chicken Council, n.d.; USDA National Agricultural Statistics Service, 2025). Values represent age-standardized incidence or prevalence per 100,000 population.

Israel’s kosher-regulated poultry system differs from chemical antimicrobial disinfection systems used in high-throughput processing environments in countries with differing PD trends; these contrasts are ecological and are intended to be interpreted within that context (World Organization for Animal Health (WOAH, formerly OIE), 2011; Israeli Ministry of Health, n.d.; Yakobson et al., 2014; Balash et al., 2025; Bourassa, 2017).

**Figure 3:**
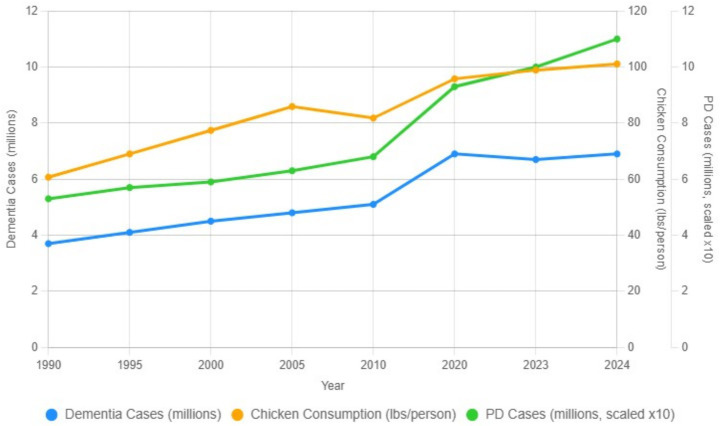
U. S. trends in dementia prevalence, Parkinson’s disease prevalence (scaled ×10), and per-capita chicken consumption, 1990–2024. Blue line: dementia cases (millions). Green line: Parkinson’s disease prevalence (millions), multiplied by 10 for visual comparison. Orange line: U. S. chicken consumption (lb/person/year, right axis). Because variables are presented in differing units and PD prevalence is scaled for visual comparison, the figure is intended to illustrate directional temporal alignment rather than quantitative association. Data sources: Global Burden of Disease Study 2021 (PD prevalence) (Steinmetz et al., 2024); Alzheimer’s Association 2024 (dementia cases) (Alzheimer’s Association, 2024); FAOSTAT 2024 and National Chicken Council data (chicken consumption) (Food and Agriculture Organization of the United Nations, 2024; National Chicken Council, n.d.). Values for 2022–2024 represent linear extrapolations. The similar upward trends of U. S. Parkinson’s disease prevalence, dementia prevalence, and per-capita chicken consumption from 1990 to 2024 are compatible with shared long-latency environmental exposures but do not establish causation. Notably, the acceleration in Parkinson’s disease and dementia prevalence emerges approximately one to two decades after major late-1990s changes in U. S. poultry-processing systems, consistent with the prodromal time course of these disorders. While aging, socioeconomic factors, and diagnostic changes influence neurodegenerative-disease estimates, U. S. trends in PD and dementia remain temporally aligned and are consistent with gut–brain inflammatory pathways (e.g., *α*-synuclein induction or neuroinflammatory priming) (Sampson et al., 2016; Foster and McVey Neufeld, 2013; Cryan and Dinan, 2012). These patterns support evaluation of whether high-throughput poultry-processing environments may represent one of several contributors to long-term disease trends.

In the United States, Parkinson’s disease and dementia prevalence (reflecting overall disease burden) have increased over the past three decades alongside per-capita chicken consumption (Figure 3). Data from the National Chicken Council (USDA Food Safety and Inspection Service, 1996), Alzheimer’s Association, and Parkinson’s Foundation (Parkinson’s Foundation, 2024) indicate that dementia prevalence increased from 3.7 to 6.9 million and Parkinson’s prevalence from 0.53 to 1.0 million, while per-capita broiler consumption rose from approximately 61 to 101 lb. per year (USDA Food Safety and Inspection Service, 1996). This temporal alignment is compatible with the long prodromal phases of these disorders but does not establish causation.

A further contrast emerges in feed-chain policy. Large-scale production systems in the United States and China permit intra-species recycling of animal by-products (e.g., rendered poultry and fish meals fed back to poultry, and feather meal in aquaculture) (FDA Center for Veterinary Medicine, 2024; U.S. Food and Drug Administration, 2008). These practices may help maintain closed-loop pathways that could support microbial persistence, particularly in high-volume production and distribution systems (USDA Economic Research Service, 2024). In contrast, Germany combines more restrictive feed practices with high-temperature rendering and oxidative/thermal sanitation, while Israel implemented post-BSE feed reforms that disrupted mammalian protein recycling (World Organization for Animal Health (WOAH, formerly OIE), 2011; Yakobson et al., 2014).

Although the ecological signal appears strongest for poultry, similar dynamics may exist in other high-volume meat systems, including pork, beef, and aquaculture. In the same period, beef and pork consumption remained comparatively stable or declined, making poultry the most internally consistent ecological signal in these national aggregates.

Statistical concordance—Poultry consumption and PD/dementia burden follow broadly similar temporal patterns across multi-year national aggregates. In selected analyses, high correlation coefficients (e.g., Pearson r ≈ 0.9–0.95) were observed. PD and dementia values represent modeled U. S. prevalence from GBD 2021 (Steinmetz et al., 2024), and poultry supply reflects FAOSTAT carcass-weight availability converted to lb./person/year (Food and Agriculture Organization of the United Nations, 2024). Beef and pork consumption remained relatively stable or declined, supporting poultry as the most internally consistent ecological signal in these comparisons. These findings are ecological and descriptive, based on unadjusted population-level data and not intended to infer individual-level causation.

External epidemiologic context—Independent studies report positive associations between meat intake and neurodegenerative outcomes. Cohort and meta-analytic analyses have linked higher consumption of processed or animal-derived meats with increased dementia risk (Tong et al., 2020; Zhang et al., 2021).

Tong et al. reported a positive ecological correlation between global meat supply—including poultry and processed meats—and dementia prevalence (r = 0.45; *p* < 0.001) (Tong et al., 2020), although these associations are ecological and subject to confounding. In a large prospective cohort from the UK Biobank, Zhang et al. found that higher processed-meat consumption was associated with increased risk of incident dementia, with evidence of a dose–response relationship (hazard ratio 1.44, 95% CI 1.24–1.67) (Zhang et al., 2021).

Figure 3 illustrates long-term U. S. trends in dementia prevalence, Parkinson’s-disease prevalence (scaled ×10), and per-capita chicken consumption from 1990–2024, showing broadly similar upward trajectories, particularly after the mid-2000s.

### Regional overlap: the “chicken belt” and neurodegenerative patterns

Major U. S. poultry-producing states (Georgia, Alabama, Mississippi, Arkansas, and North Carolina) account for roughly half of national broiler production (USDA National Agricultural Statistics Service, 2025; USDA Economic Research Service, 2024). These regions overlap with areas reporting relatively elevated PD and dementia burden relative to national averages in available GBD estimates (Steinmetz et al., 2024; Alzheimer’s Association, 2024). Portions of the Midwest overlapping with the historically recognized “PD belt” show similar patterns.

However, because poultry products from high-volume facilities are distributed nationwide, any exposure signal would not be limited to production regions. Instead, exposure could be “peppered” across broad downstream populations through modern distribution networks. Multiple socioeconomic, demographic, and healthcare-access factors also influence regional disease rates, so these spatial associations must be interpreted with caution. The spatial association between intensive poultry-processing areas and higher neurodegenerative burden is noted here as an area that may warrant further investigation into environmental and microbial exposures (Steinmetz et al., 2024; Alzheimer’s Association, 2024; USDA National Agricultural Statistics Service, 2025; USDA Economic Research Service, 2024).

## Mechanistic hypotheses relevant to PD and dementia

### Microbial persistence and biofilm adaptation

Certain bacteria present in food-processing environments, including organisms such as *Brachyspira* spp., can form structured biofilms that enhance tolerance to environmental stressors, including heat, desiccation, and disinfection (Bridier et al., 2011; Giaouris et al., 2014; Calabrese and Mattson, 2017). Surveys conducted in the United Kingdom during the 1990s and 2000s reported *Brachyspira* prevalence in poultry flocks ranging from 46 to 71% (Burch et al., 2006). Several poultry-associated *Brachyspira* species, including *B. pilosicoli*, are recognized zoonotic organisms capable of colonizing the human intestinal tract and forming adherent, biofilm-like structures on mucosal surfaces, supporting their relevance to long-latency exposures in food-chain systems (Trott et al., 1997; Jensen et al., 2001; Verlinden et al., 2012). Recent studies demonstrate that biofilm-associated bacteria in these environments can persist under sanitation pressures (Bridier et al., 2011; Giaouris et al., 2014; Calabrese and Mattson, 2017; Galié et al., 2018), however, the presence and persistence of spirochetes or related taxa remain insufficiently characterized. Viable *Brachyspira* spp. have also been detected on retail poultry products, supporting the possibility of post-processing persistence and downstream exposure (Verlinden et al., 2012).

Biofilm-associated bacteria differ from free-floating (planktonic) cells in their response to disinfection, as the extracellular matrix can limit disinfectant penetration, particularly under high organic-load conditions. As a result, chemical disinfectants that are effective under controlled conditions may be less effective in complex processing environments characterized by high organic load, surface irregularities, and established biofilms (Bridier et al., 2011; Giaouris et al., 2014; Calabrese and Mattson, 2017).

Repeated sublethal exposure to disinfectants such as chlorine-based compounds or peracetic acid (PAA)—commonly used in poultry-processing environments—has been associated with adaptive stress responses, including increased extracellular-matrix production and enhanced tolerance. Once established, biofilms may persist through sanitation cycles and contribute to recontamination and recirculation across processing surfaces and downstream environments (Bridier et al., 2011; Giaouris et al., 2014; Calabrese and Mattson, 2017).

Routine regulatory surveillance programs, including those of the USDA Food Safety and Inspection Service and U. S. Food and Drug Administration, focus on established foodborne pathogens such as *Salmonella*, *Campylobacter*, and indicator organisms (e.g., generic *E. coli*), as reflected in current inspection and HACCP-based testing frameworks (USDA Food Safety and Inspection Service, 1996; USDA Food Safety and Inspection Service, 2023), and do not typically include targeted screening for spirochetes or other fastidious biofilm-associated organisms.

**Figure 4:**
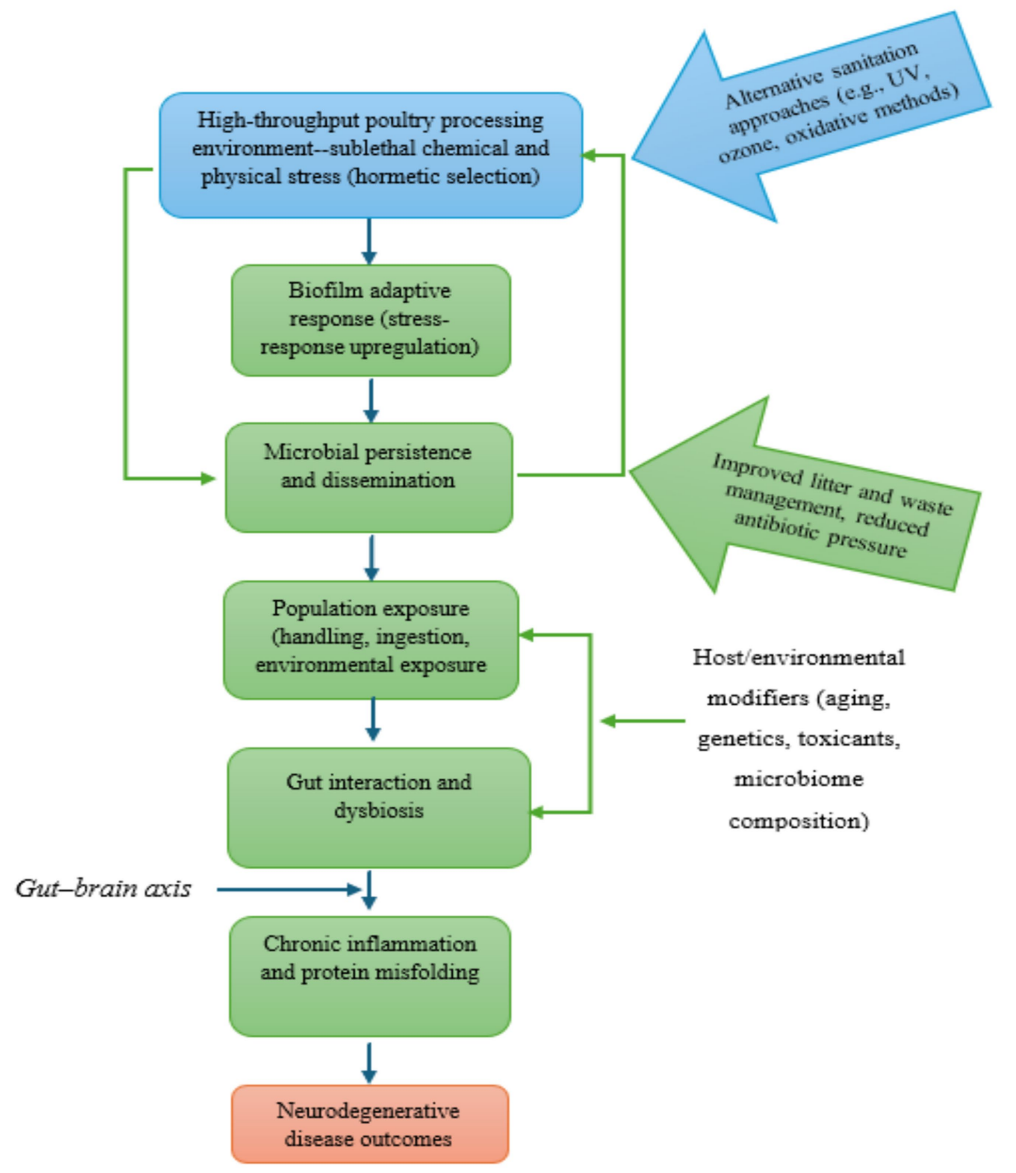
Conceptual schematic of a spirochete persistence cycle and pathways of microbial persistence across poultry production and human exposure.

Figure 4 illustrates this process.

### Population-level exposure pathways

The food chain encompasses animal production and processing, distribution, retail handling, and consumption. Exposure pathways extend beyond occupational settings to the general population.

Individuals may encounter microbial agents through handling of raw poultry products, cross-contamination within domestic or commercial kitchens, and ingestion. Environmental interfaces—including wastewater, surface contamination, and animal feed—may also contribute to indirect exposure.

Evidence of post-processing persistence is supported by studies demonstrating that biofilm-associated microorganisms can survive sanitation processes and persist in food-processing environments (Bridier et al., 2011; Giaouris et al., 2014). Such persistence indicates that certain organisms may remain beyond initial processing and enter consumer-facing environments, supporting exposure through handling, preparation, and consumption of poultry products.

These routes do not require direct contact with production environments and support widespread, low-level exposure over time. Such repeated exposures may be relevant in long-latency conditions such as Parkinson’s disease and dementia, where cumulative environmental influences interact with aging and host susceptibility.

Poultry-processing practices are not uniform across facilities or regions, and variability in disinfection methods, throughput, and operational controls has been documented (National Chicken Council, n.d.; USDA Food Safety and Inspection Service, 1996; USDA Food Safety and Inspection Service, 2023). These differences may influence microbial persistence and contribute to heterogeneous exposure across the food system. Within this context, food-chain-mediated exposure is proposed as one contributor among several rather than a sole determinant.

Persistence mechanisms help explain how repeated exposure may translate into biological effect. Biofilm-forming spirochetes exposed to sublethal stressors may undergo adaptive responses that enhance survival in processing and environmental reservoirs (Bridier et al., 2011; Giaouris et al., 2014; Calabrese and Mattson, 2017).

Ingested organisms may influence inflammatory pathways via the gastrointestinal environment, affecting vagal signaling and immune activation—key elements of the gut–brain axis (Sampson et al., 2016; Foster and McVey Neufeld, 2013; Cryan and Dinan, 2012). Over time, such responses have been linked to protein misfolding processes, including *α*-synuclein and *β*-amyloid, providing a plausible link between persistent gastrointestinal microbes and neurodegenerative pathology (Sampson et al., 2016; Foster and McVey Neufeld, 2013; Cryan and Dinan, 2012).

The proposed pathway can be conceptualized as: persistent organisms within high-throughput processing environments → dissemination through food handling, consumption, and environmental interfaces → entry into the gastrointestinal tract → repeated exposure → interaction with host factors → chronic inflammation and downstream neurodegenerative processes.

### Gut-brain axis and protein misfolding

The gut–brain axis provides a biologically plausible pathway by which environmental microbes may influence central nervous system function. Experimental and clinical studies indicate that gut-associated microbiota and their metabolites can influence *α*-synuclein aggregation, neural signaling, immune activation, and inflammatory pathways, including vagal and enteric signaling (Sampson et al., 2016; Foster and McVey Neufeld, 2013; Cryan and Dinan, 2012).

In Parkinson’s disease, α-synuclein aggregation has been observed in the enteric nervous system and may precede central nervous system involvement, consistent with a peripheral origin (Sampson et al., 2016). Similar inflammatory and microbiome-associated mechanisms have been implicated in broader neurodegenerative processes, including *β*-amyloid and tau pathology (Foster and McVey Neufeld, 2013; Cryan and Dinan, 2012).

Persistent microbial exposure is proposed as one upstream stimulus that may contribute to or amplify these processes. Supporting this concept, exposure to the bacterial amyloid protein curli (produced by certain enteric bacteria) has been shown to enhance *α*-synuclein aggregation in animal models via cross-seeding (Chen et al., 2016). Although direct evidence linking *Brachyspira* or other spirochetes to this specific process is currently lacking, spirochetes share several relevant biological features with organisms known to produce functional amyloids, including the ability to form biofilms, persist chronically in mucosal environments, and sustain low-grade inflammation (Miklossy, 2011; Trott et al., 1997; Jensen et al., 2001; Verlinden et al., 2012).

Chronic inflammation triggered by such persistent organisms can promote oxidative stress, cytokine release, and impaired protein clearance, conditions known to favor *α*-synuclein misfolding and prion-like propagation (Brundin et al., 2010). In this integrative model, resilient spirochetes or related taxa encountered through food-chain exposure could act as one repeated, low-level trigger that interacts with host susceptibility factors via the gut–brain axis. This perspective is consistent with misfolded proteins acting as downstream amplifiers within a chronic inflammatory milieu rather than solely initiating agents (Brundin et al., 2010), while remaining compatible with toxicant and oxidative-stress models.

This model is intended as an integrative framework linking established microbiome–brain interactions with a potential environmental exposure pathway, rather than a demonstration of direct causation.

### BSE and vCJD archives as opportunities for re-examination

Classical prion theory was developed to explain the transmissible spongiform encephalopathies, including bovine spongiform encephalopathy (BSE) and variant Creutzfeldt–Jakob disease (vCJD) (Prusiner, 1998). When these models were established, methods for detecting fastidious, biofilm-forming, or low-abundance microorganisms were limited. Archived materials from BSE and vCJD therefore provide an opportunity for re-examination using contemporary molecular and histologic techniques.

Neuropathologic features associated with these disorders—such as spongiform change, gliosis, and amyloid deposition—have also been described in certain chronic inflammatory and microbial conditions (Allen et al., 2016; Miklossy, 2011; Miklossy, 2015). This does not imply a shared etiology and is not specific to spirochetes, but reflects a broader question of whether persistent, difficult-to-detect microorganisms may coexist with or modulate, established protein-misfolding pathways. Prion infectivity is concentrated primarily in central nervous system tissue, whereas many microbes, including spirochetes, naturally colonize the intestinal tract and lymphoid tissues—the materials that were historically rendered and recycled in feed during the BSE era (FDA Center for Veterinary Medicine, 2024; U.S. Food and Drug Administration, 2008; U.S. Department of Agriculture Animal and Plant Health Inspection Service, 2024).

Formalin-fixed, paraffin-embedded brain and lymphoreticular specimens from vCJD cases and UK prevalence surveys may be suitable for re-analysis with modern methods (Miklossy, 2011; Riviere et al., 2002). Such re-examination represents a low-cost and underexplored opportunity to determine whether persistent bacteria were present but overlooked in the original investigations. Although sensitivity may be reduced by DNA fragmentation and cross-reactivity among spiral-shaped taxa, detection of microbial DNA or ultrastructural features, while requiring careful contamination control and orthogonal validation, could be informative.

The BSE episode illustrates how feed-chain recycling can amplify agents capable of contributing to neurological risk. Re-analysis of these archives does not challenge the established prion framework, which remains the primary explanatory model for BSE/vCJD, but could clarify whether microbial cofactors played an under-appreciated role. This historical parallel supports applying similar molecular scrutiny to questions of microbial persistence in modern high-throughput animal-production and processing systems.

### Integrative perspective

These mechanisms can be conceptualized within a shared microbe–inflammation–protein misfolding axis. The proposed model integrates several principal theories (Table 2).

**Table 2:** Principal theories of neurodegeneration and their relationship to the microbial-persistence model.

Major theory	Core premise	Relationship to microbial-persistence model
Infection hypothesis	Chronic pathogens (including spirochetes) initiate sustained inflammation	Proposed as one initiating factor.
Gut-brain axis	Enteric microbiota and immune signaling influence brain pathology	Provides the physiologic conduit linking infection to neurodegeneration
Prion-like propagation	Misfolded proteins spread pathology cell-to-cell	Represents downstream amplification of processes related to inflammatory or microbial triggers (Chen et al., 2016)
Toxicant and oxidative stress	Chemical or metabolic stress causes neuronal injury	May arise as a secondary effect of microbial metabolism or chemical hormesis

Established mechanistic theories can be integrated within an ecological context, in which microbial persistence is proposed as an upstream contributor acting alongside aging, environmental exposures, metabolic influences, and genetics. Temporal and geographic alignment between industrial poultry expansion and rising neurodegenerative burdens (Steinmetz et al., 2024; Food and Agriculture Organization of the United Nations, 2024; National Chicken Council, n.d.) is compatible with further evaluation of microbial-persistence pathways, including spirochetes (Table 3).

**Table 3:** Comparative strength of evidence for major neurodegenerative triggers.

Proposed primary trigger	Mechanistic focus	Representative association (approx.)	Key supporting sources	Relationship to spirochetal-persistence model
Aging	Cellular senescence, mitochondrial decline, oxidative stress	Risk increases with age, particularly after age 60	Well-established epidemiologic finding	Age may increase vulnerability to persistent microbial exposure and reduced clearance capacity
Genetic susceptibility	Mutations affecting protein handling or inflammation (LRRK2, SNCA, APOE-ε4)	OR ≈ 2–5	Brundin et al. (2010)	Genetic variation may modulate inflammatory response to chronic infection
Environmental toxicants (e.g., pesticides)	Pesticide exposure	OR ≈ 1.5–2.0 (meta-analytic ranges)	Van Maele-Fabry et al. (2012)	Toxicants may contribute to inflammatory processes and may influence selection for more resilient microbial populations
Gut-brain axis	Microbiota–brain signaling and vagal pathways	Supported by experimental and observational evidence	Sampson et al. (2016), Foster and McVey Neufeld (2013)	Spirochetes are consistent with this pathway as one class of chronic gut-associated organisms contributing to inflammation
Prion-like propagation	Cell-to-cell spread of misfolded α-synuclein or *β*-amyloid	Supported by mechanistic studies and pathology	Brundin et al. (2010)	Chronic microbial inflammation may contribute to processes related to protein misfolding that subsequently propagate
Chronic infections	Chronic infections (including spirochetes and gut-associated pathogens)	Associations reported in observational and mechanistic studies	Allen et al. (2016), Miklossy (2011)	Spirochetes share features of infectious agents, including neurotropism and latency, as described in prior studies.
Spirochetal persistence (proposed)	Chronic biofilm-forming infection (*Brachyspira, Borrelia, Treponema*)	Temporal and geographic alignment; consistent ecological correlations observed in multiple datasets (population-level, non-causal)	Miklossy (2011), Riviere et al. (2002), Tong et al. (2020)	Proposed as an integrative ecological model linking microbial, inflammatory, gut-brain, toxicant, and prion-like pathways

Spirochetes and other chronic microbial agents have been proposed in prior neurodegenerative models, often with mixed or inconclusive evidence. The present hypothesis differs by emphasizing a population-level ecological exposure pathway grounded in food-system dynamics and amenable to evaluation across environmental, clinical, and experimental settings, rather than relying solely on isolated pathogen detection.

## Discussion and public-health implications

### Synthesis of findings

The temporal alignment between the expansion of high-throughput poultry processing and rising burdens of Parkinson’s disease and dementia across multiple regions (Steinmetz et al., 2024; Food and Agriculture Organization of the United Nations, 2024; National Chicken Council, n.d.) is notable. Contrasting trends in countries with different sanitation and feed-chain policies further support evaluation of whether microbial persistence in food systems may contribute to long-latency neurodegenerative risk. These patterns align with the known prodromal phases of both disorders. However, the associations remain ecological and are subject to multiple confounders, including diagnostic improvements, population aging, and other environmental exposures. The mechanistic framework presented here remains based on convergent but indirect evidence and requires direct experimental validation.

### Policy and research implications

If persistent biofilm-forming organisms contribute to neurodegenerative risk, prevention efforts could usefully include evaluation of approaches that limit microbial adaptation and biofilm persistence, rather than relying predominantly on intensified chemical disinfection (Bridier et al., 2011; Giaouris et al., 2014; Calabrese and Mattson, 2017). Alternative sanitation technologies—such as ozone, UV-C, cold atmospheric plasma, and non-chemical methods (e.g., kosher salting and rinsing) (Israeli Ministry of Health, n.d.; Giménez et al., 2024; Niveditha et al., 2021)—merit further study in high-throughput poultry-processing environments.

Antibiotic stewardship in poultry production also warrants consideration, as sub-inhibitory exposure can promote biofilm formation and microbial persistence (Bourassa, 2017). Regional differences in sanitation practices and feed-chain policies provide natural experiments for understanding how microbial ecology may influence long-term exposure risk [World Organization for Animal Health (WOAH, formerly OIE), 2011; Israeli Ministry of Health, n.d.; Yakobson et al., 2014; Balash et al., 2025; Fink et al., 2025; Rommel et al., 2025a,b; Lutski et al., 2022, Hou and Yang, 2025; USDA National Agricultural Statistics Service, 2025].

### Testable predictions

Key testable predictions include: (1) detection of resilient spirochetes or related taxa after sanitation in processing environments or retail products; (2) higher prevalence or burden of spirochetal markers in gastrointestinal or cerebrospinal fluid samples from PD and dementia patients compared with controls; (3) induction of neuroinflammatory and protein-misfolding responses in experimental models of chronic exposure; and (4) measurable stabilization of neurodegenerative trends following reductions in microbial persistence, after appropriate lag periods and adjustment for confounders. These predictions are falsifiable. Convergent findings across environmental, clinical, and experimental studies would strengthen the case for microbial persistence as a contributing factor, while consistent negative results would weaken it.

### Broader significance

Reducing microbial persistence in food systems could have implications beyond neurodegeneration, including improved antimicrobial stewardship and food safety. The persistence-infection hypothesis offers one testable ecological lens through which modern industrial practices may intersect with long-latency neurological disease.

## Conclusions and future directions

### Summary of evidence

The evidence reviewed supports a testable ecological model in which microbial persistence in modern food systems may interact with aging, host susceptibility, and established protein-misfolding pathways to contribute to long-latency neurodegenerative risk. Across observational, mechanistic, and comparative domains, persistent microbial exposures—particularly biofilm-forming organisms—emerge as plausible but currently unproven upstream contributors. Direct molecular validation remains essential.

## Limitations and future work

### Ecological inference

The associations presented are strictly ecological and population-level. They cannot establish individual-level causation and are susceptible to the ecological fallacy. Observed temporal and geographic patterns may be driven by changes in diagnostic practices, improved case ascertainment, population aging, healthcare access, and other unmeasured environmental or lifestyle factors.

### Sampling biases and data limitations

Neurodegenerative disease estimates from the Global Burden of Disease study rely on modeling and surveillance systems that vary in quality and completeness across regions and time. Meat supply data reflect availability rather than actual individual consumption or exposure routes.

### Uncontrolled confounders

Multiple established risk factors for Parkinson’s disease and dementia—including aging, genetic susceptibility, pesticides, air pollution, metals, viral infections, and broader dietary patterns—were not controlled for and may contribute independently or interactively to the observed trends.

### Molecular and microbiological evidence gap

Direct evidence of spirochetal or other fastidious microbial persistence in post-sanitation poultry products, processing environments, or clinical samples from patients with Parkinson’s disease or dementia is currently lacking. Organisms such as *Brachyspira* spp. are difficult to detect because of their biofilm-forming capacity and the technical limitations of culture and sequencing methods in complex environmental and tissue matrices. Archived formalin-fixed, paraffin-embedded tissues present additional challenges, including DNA fragmentation and the risk of contamination, which necessitate rigorous controls and orthogonal validation methods in any future analyses.

It should also be noted that while certain histopathological and molecular studies have reported the presence of spirochetes (including *Treponema* species) in brain tissue of patients with Alzheimer’s disease (Miklossy, 2011; Miklossy, 2015; Riviere et al., 2002), these observations remain controversial. Findings have been mixed across studies, with some failing to replicate detections or attributing positive results to postmortem changes, contamination, or secondary colonization rather than primary causation (Gadila et al., 2021; Frölich, 2020). Broader microbiome research in Parkinson’s disease and dementia consistently shows associations with dysbiosis and neuroinflammation, but has not established persistent food-chain spirochetes (such as poultry-associated *Brachyspira*) as a major upstream driver (Fang et al., 2020; Lubomski et al., 2022; Brown, 2024). The present hypothesis therefore positions chronic microbial persistence as one plausible but currently unproven ecological contributor that warrants rigorous empirical testing alongside other microbial, inflammatory, and environmental factors.

### Overall interpretation

Collectively, these limitations indicate that the microbial-persistence hypothesis remains speculative and strictly hypothesis-generating. The ecological patterns are compatible with—but do not prove—a contribution from food-chain microbial dynamics to long-latency neurodegeneration.

### Future directions

Future empirical studies—including environmental metagenomics, case–control microbiome profiling, experimental models of chronic low-level exposure, and re-analysis of archived tissues—will be essential to test, refine, or refute this framework.

The persistence-infection hypothesis offers a testable conceptual framework through which microbial adaptation in high-throughput food systems may relate to long-latency neurodegenerative disease risk. Clarifying the role of persistent microbial exposures will require coordinated, multidisciplinary investigation. Until then, the ideas presented here are intended to stimulate rigorous empirical investigation.

## Data Availability

Publicly available datasets were analyzed in this study. This data can be found here: Global Burden of Disease (GBD) Study 2021 (PD and dementia estimates): https://ghdx.healthdata.org/gbd-results-tool FAOSTAT Food Balance Sheets (poultry/meat consumption data): https://www.fao.org/faostat/en/#data/FBS National Chicken Council (U. S. per-capita poultry consumption trends): https://www.nationalchickencouncil.org/statistic/per-capita-consumption-poultry/ USDA Economic Research Service (Livestock & Meat Domestic Data): https://www.ers.usda.gov/data-products/livestock-meat-domestic-data/.
